# GC-MS identification of a lacthydrazide-class siderophore produced by *Streptomyces sp*. VITGV100: its antimicrobial activity and molecular docking

**DOI:** 10.5114/bta/224355

**Published:** 2026-09-21

**Authors:** Madhuri Mukindrao Moon, John Godwin Christopher

**Affiliations:** School of BioSciences and Technology, Vellore Institute of Technology, VIT University, Vellore, Tamil Nadu, India

**Keywords:** *Streptomyces*, GC-MS analysis, antimicrobial compounds, molecular docking

## Abstract

**Background:**

The bacterial genus *Streptomyces* comprises well-known producers of bioactive compounds, mainly antibiotics. Specifically, the strain *Streptomyces* sp. VITGV100 shows promise as a potent antimicrobial source. This study investigates the activation of cryptic biosynthetic genes within this strain through chemical elicitation to amplify the production of secondary metabolites.

**Materials and methods:**

To achieve this, *Streptomyces* sp. VITGV100 was cultured in nutrient broth supplemented with 0.5% dimethyl sulfoxide (DMSO) to elicit metabolic changes over 7, 14, and 21 days. The resulting crude extracts were screened for antimicrobial activity against four human pathogens: *Escherichia coli* (Microbial Type Culture Collection and Gene Bank [MTCC] 1687), *Pseudomonas aeruginosa* (MTCC 3541), *Bacillus subtilis* (MTCC 2756), and *Staphylococcus aureus* (MTCC 737). Bioactive fractions were characterized by gas chromatography-mass spectrometry (GC-MS). Finally, identified compounds, namely lacthydrazide, were docked against target proteins: *E. coli* Z-ring-associated protein (5IMJ), *P. aeruginosa* OXA10 (4WZ5), *S. aureus* TyrRS (1JIJ), and *B. subtilis* DLTA (3E7X).

**Results:**

The 7-day crude extract exhibited the strongest antimicrobial activity, yielding a maximum inhibition zone of 31 mm against *E. coli* at 100 μg/ml and a minimum zone of 9 mm against *B. subtilis* at 25 μg/ml. GC-MS analysis revealed 45 distinct peaks in the included extracts versus 30 in controls, identifying lacthydrazide as a primary compound. Molecular docking confirmed the strong binding affinity of lacthydrazide to the target proteins, supporting its antimicrobial potential.

**Conclusions:**

*Streptomyces* sp. VITGV100, elicited with 0.5% DMSO, effectively activated cryptic genes to synthesize potent antibacterial compounds. These findings position this strain as a valuable resource for discovering novel antimicrobials.

## Introduction

The genus *Streptomyces* comprises filamentous Gram-positive bacteria belonging to the phylum *Actinomycetes* (Al-Ansari et al. 2019). It represents one of the most prolific producers of antibiotics, accounting for nearly two-thirds of all known antimicrobial compounds (Alam et al. 2022). These organisms synthesize diverse secondary metabolites, including antibacterial, antifungal, anticancer, and antiviral agents (Ikhrami et al. 2021). Concurrently, the World Health Organization (WHO) classifies antimicrobial resistance among the top 10 global public health threats (Salam et al. 2023). Although frequently overlooked in the Western Hemisphere, diseases such as tuberculosis and plague exert a severe impact on individuals and society (Merbs 1992). To address this, the WHO published a list of multidrug-resistant pathogens to prioritize microorganism-specific antibiotic development (Rello et al. 2019). The rapid dissemination of antibiotic-resistant microorganisms underscores the urgent need to discover and develop novel antimicrobial compounds (Widmer 2022).

In *Streptomyces*, secondary metabolite production is governed by clusters of closely spaced protein-coding genes, known as biosynthetic gene clusters (BGCs) (Lee et al. 2021). Decade-long advancements in wholegenome sequencing have uncovered numerous cryptic biosynthetic genes capable of synthesizing unknown secondary metabolites (Veilumuthu et al. 2022). Although certain genes are active under standard laboratory conditions, others remain inactive and are categorized as cryptic or silent (Hoskisson and Seipke 2020). Consequently, antiSMASH analysis is crucial for identifying and characterizing these BCGs responsible for the production of secondary metabolites. To activate these silent gene clusters, various strategies have been developed, including heterologous expression (Matsuda et al. 2017), co-culture (Onaka 2017), ribosome engineering (Zhu et al. 2019), and elicitation by biological (El-Hawary et al. 2023), chemical (Zong et al. 2022), and physiological agents (Tyurin et al. 2018).

Among these, chemical elicitors particularly excel at enhancing metabolite production by triggering the expression of silent genes (Moon and Christopher 2025). Using dimethyl sulfoxide (DMSO) as a chemical elicitor can stimulate latent biosynthetic pathways in *Streptomyces* sp. VITGV100, thereby driving the production of novel antimicrobial metabolites.

Computer-aided drug discovery complements experimental approaches by predicting molecular interactions, refining lead compounds, and assessing biological activity (Chaudhary and Mishra 2016). Consequently, computational platforms have emerged as pivotal tools in the search for drugs derived from natural sources. This approach enables potential drug target identification, high-throughput screening, lead optimization, and side effect evaluation (Segall and Barber 2014). Synergizing experimental and computational approaches allows elucidating molecular interactions within protein binding sites, facilitating the development of more effective, selective therapies (Pagadala et al. 2017).

This study evaluated the antimicrobial potential of *Streptomyces* sp. VITGV100 by employing DMSO as an elicitor to activate cryptic gene clusters and analyzing the resulting secondary metabolites, with a focus on antimicrobial compounds. We also identified and characterized novel antimicrobial compounds produced by this strain. Furthermore, molecular docking simulations against key bacterial target proteins were conducted to elucidate their mechanisms of action and validate their antimicrobial properties.

## Materials and methods

### Streptomyces sp. VITGV100 (MCC 4961)

A pure culture of *Streptomyces* sp. VITGV100 (MCC 4961) was maintained on International Streptomyces Project medium 2 and incubated at 30°C for 5 days to yield log-phase cells for subsequent experiments.

### Elicitation and extraction

*Streptomyces* sp. VITGV100 were cultured in six flasks containing 300 ml of nutrient broth supplemented with 0.5% (v/v) DMSO. A control without DMSO was also included. The cultures were incubated at room temperature on an orbital shaker at 150 rpm for 7, 14, and 21 days, respectively. Following incubation, the cultures were centrifuged at 8000 rpm for 20 min to separate the cell biomass. Secondary metabolites were extracted from the broth using a two-phase solvent system with ethyl acetate (1 : 1). A rotary evaporator (model RE100-Pro) was used to concentrate the organic extracts at 54°C and 80 rpm. The concentrated crude extract was further analyzed by gas chromatography-mass spectrometry (GC-MS).

### Antimicrobial activity screening

The antimicrobial potency of the crude extract was evaluated against four human pathogenic bacteria: *Staphylococcus aureus* (Microbial Type Culture Collection and Gene Bank [MTCC] 737), *Pseudomonas aeruginosa* (MTCC 3541), *Bacillus subtilis* (MTCC 2756), and *Escherichia coli* (MTCC 1687). Antibacterial assays were conducted in the crude extract via the agar well diffusion method. Briefly, 25 μl of the bacterial suspension was spread uniformly onto nutrient agar plates. Wells were then punched with a sterile 6-mm cork borer and filled with 100 μl of the crude extract at four different concentrations (25, 50, 75, and 100 μg/ml). The plates were preincubated at 4°C for 24 h, followed by incubation at 37°C. The resulting inhibition zones of the test microorganisms around the wells were measured and recorded (Khoshmaram et al. 2024).

### GC-MS analysis

The crude extract was analyzed using a GC-MS system (Thermo Scientific, Waltham, MA) comprising a Trace GC Ultra coupled with an ISQ Single Quadrupole Mass Spectrometer. Separation was carried out on a TG-5MS fused silica capillary column (30 m × 0.25 mm × 0.1 mm film thickness). Electron ionization at 70 eV was employed for metabolite detection. Helium served as the carrier gas at a constant flow rate of 1 ml/min. The injector and MS transfer line temperatures were maintained at 280°C. The oven temperature program commenced at 50°C, increased to 150°C within 7 min and then to 270°C in 2 min, and finally reached 310°C in 3.5 min. The analytical sensitivity of the method was confirmed by determining the limits of detection and quantification based on signal-to-noise ratios of 3 : 1 and 10 : 1, respectively. Calibration curves displayed strong linearity, with correlation coefficients (R^2^) exceeding 0.99. Reproducibility was verified via repeated analyses of a standard sample under identical conditions, yielding a relative standard deviation below 5%.

### Molecular docking

#### Binding site prediction

The selection of binding sites was guided by previous research and the presence of co-crystallized ligands in the obtained protein structures (Heo et al. 2014). Following the identification of binding sites, the coordinates for the binding pocket of each target protein were determined using the Define and Edit Binding Site tool in BIOVIA Discovery Studio.

### Ligand preparation

The annotated compound lacthydrazide was acquired in .pdb format from PubChem (Fu et al. 2015). To facilitate docking, polar hydrogens and Gasteiger charges were incorporated, and the compounds were then converted to PDBQT format using AutoDock software.

### Protein preparation

The protein targets, namely *E. coli* Z-ring associated protein (Protein Data Bank [PDB] ID: 5IMJ), *P. aeruginosa* OXA10 (PDB ID: 4WZ5), *S. aureus* TyrRS (PDB ID: 1JIJ), and *B. subtilis* DLTA non-ribosomal peptide synthetase (NRPS) adenylation domains (PDB ID: 3E7X), were sourced from the Research Collaboratory for Structural Bioinformatics (RCSB) PDB. Following water removal, the structures were optimized with Kollmann charges, and polar hydrogens were added using AutoDock tools before being saved in PDBQT format. To facilitate docking, a three-dimensional affinity grid box measuring 40 × 40 × 40 Å was generated around the binding sites of the target proteins and centered more on crucial residues predicted to interact with potent metabolites.

### Molecular docking and validation

Virtual screening was carried out using AutoDock tools version 1.5.7 to dock the identified compounds into the binding sites of the target proteins. This approach resulted in a collection of poses, sorted by ∆G, which represents the expected binding energy in kcal/mol. Subsequently, the presence of chemicals within the target proteins was examined using BIOVIA Discovery Studio. The optimum pose was identified by evaluating hydrogen bonding and hydrophobic interaction with binding residues.

### Statistical analyses

All data are presented as the mean ± standard error of the mean. Statistical analysis was performed using a two-way analysis of variance (ANOVA) in GraphPad Prism Version 10. Differences were considered statistically significant at *p* < 0.05.

## Results and discussion

Actinomycetes are known for producing diverse bioactive compounds that play crucial roles in combating infectious diseases (Dimri et al. 2020). Among them, *Streptomyces* species remain the most significant producers of clinically valuable metabolites. In this study, *Streptomyces* sp. VITGV100 was analyzed as a promising candidate for discovering novel antimicrobial compounds due to its potential to synthesize biologically active secondary metabolites. A recent genome analysis conducted via antiSMASH 6.0 revealed 35 BGCs (Veilumuthu et al. 2022) implicated in the production of secondary metabolites. Among these, certain clusters demonstrated low similarity to known antimicrobial compounds, with approximately 9% (three BGCs) predicted to govern siderophore biosynthesis (Veilumuthu et al. 2022).

This study aimed to activate BGCs using DMSO as a chemical elicitor to induce the synthesis of unique bioactive compounds. The resulting compounds exhibited pronounced zones of inhibition against both Gram-positive and Gram-negative bacteria. These bacteria help to evaluate the efficacy of natural antimicrobial products due to their widespread prevalence, unique cell topology, and clinical relevance.

### Antimicrobial activity

*Streptomyces* sp. VITGV100 demonstrated a broad spectrum of antibacterial properties (Table 1). The pathogens *E. coli, P. aeruginosa, B. subtilis*, and *S. aureus* displayed differential sensitivity to the antibacterial activity of crude extracts. The highest percentage of inhibition was observed in the 7-day extract elicited with 0.5% DMSO. The largest inhibition zone was achieved against *E. coli* (31 mm), followed by *P. aeruginosa, B. subtilis* (30 mm), and *S. aureus* (29 mm). Conversely, lower inhibitory activity was recorded for extracts from the other incubation days. On days 14 and 21, the maximum zone of inhibition decreased to 20 and 21 mm for *E. coli* and *P. aeruginosa*, respectively, while the minimum zones of inhibition were measured at 9 and 10 mm for *B. subtilis* and *S. aureus*, respectively. In the positive control treated with tetracycline, the maximum zone size was 26 mm for *E. coli* and *B. subtilis*, and the minimum zone size was 20 mm for *P. aeruginosa*. Specifically, compared to the 7-day extract, the tetracycline-treated extract demonstrated less inhibition. The negative control comprising ethyl acetate revealed no zone of inhibition.

**Table 1:** Zones of inhibition exhibited by pathogens across different incubation days and controls

Incubation days	Zone of inhibition of selective human pathogen (mm)															
	*Escherichia coli*				*Pseudomonas aeruginosa*				*Staphylococcus aureus*				*Bacillus subtilis*			
	25	50	75	100	25	50	75	100	25	50	75	100	25	50	75	100
7	25	27	28	31	20	25	28	30	22	25	27	29	23	26	28	30
14	14	17	18	20	12	14	15	19	11	13	14	19	10	12	15	18
21	11	12	13	16	12	15	19	21	10	13	17	19	09	10	13	15
Tetracycline	21	22	23	26	20	22	23	24	22	24	24	25	22	24	25	26
Ethyl acetate	–	–	–	–	–	–	–	–	–	–	–	–	–	–	–	–

In the present study, the size of the inhibition zone served as the primary criterion for assessing antimicrobial activity against the selected pathogens. Notably, pronounced zones of inhibition were observed, as depicted in Figure 1. The 7-day extract exhibited the largest zone of inhibition at 31 mm against *E. coli*, indicating potent antimicrobial activity. Conversely, the smallest inhibition zone of 9 mm was observed against *B. subtilis*, indicating relatively weaker activity against this pathogen. A previous study reported that *Streptomyces* sp. VITGV100 yielded a maximum 22-mm zone of inhibition against *E. coli* and a minimum 8-mm zone against *B. subtilis* (Veilumuthu et al. 2022). Collectively, our findings demonstrate that strain VITGV100 possesses substantial antimicrobial activity against all four tested pathogens. This inhibitory effect may be driven by L-leucine, N-cyclopropylcarbonyl-, methyl ester (Ndwigah et al. 2015), 2-fluorobenzoic acid heptadecyl ester (Fanele and Ndlovu 2023), methyl tris(trimethylsiloxy)silane (Manikandan et al. 2019), cis-4-decene (Strobel et al. 2017), or other antimicrobial compounds that can impede bacterial DNA or RNA synthesis, cell wall formation, or protein synthesis. Additional research is required to validate these mechanisms. Strikingly, the crude extract demonstrated a greater zone of inhibition than the standard drug tetracycline (26 mm). Thus, these crude extracts represent a promising reservoir of antibiotics. The effects of the DMSO elicitor, as well as varying incubation durations, on antibacterial activity were examined using a two-way ANOVA. Significantly larger zones of inhibition were seen in several extracts, particularly the 7-day incubation extract, and the differences were statistically significant (*p* > 0.05) (Supplementary Figures 1–4).

**Figure 1:**
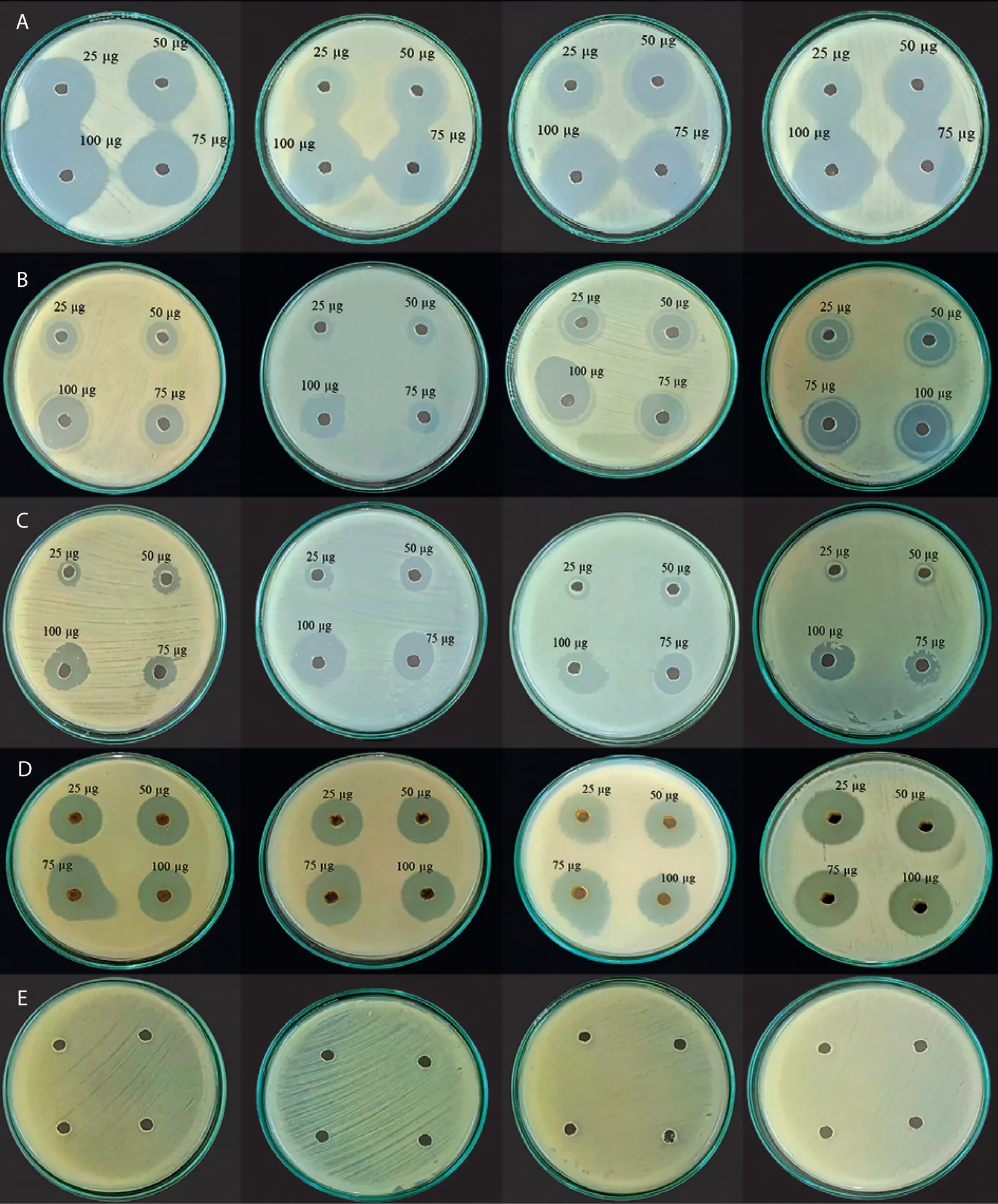
Antibacterial activity of *Streptomyces* sp. VITGV100 culture extract against *Escherichia coli, Pseudomonas aeruginosa, Staphylococcus aureus*, and *Bacillus subtilis* at varying concentrations (25, 50, 75, 100 μg/ml) by well diffusion method: (**A**) denotes the inhibition zone for the 7^th^ day, (**B**) the inhibition zone for the 14^th^ day, (**C**) the inhibition zone for the 21^st^ day, **(D)** the positive control tetracycline, and (**E**) denotes the negative control, ethyl acetate

### GC-MS analysis

The ethyl acetate extract of *Streptomyces* sp. VITGV100 was subjected to GC-MS analysis to identify the constituent bioactive compounds. Compound identification was confirmed based on peak areas and molecular weights in the spectral data. These two characteristics are directly proportional to the concentration of the compound in the extract. The GC-MS profiling of the ethyl acetate culture extracts from days 7, 14, and 21 elicited with 0.5% DMSO revealed the presence of 45 distinct peaks in each extract (Figure 2A–C), whereas their respective controls showed only 30 peaks (Figure 3A–C). The extract from the 7-day-old culture contained 14 unique compounds, representing the peak area percentages detailed in Table 2. Similarly, the extract from the 14-day-old culture yielded 14 unique compounds (Table 3), and the 21-day-old culture extract had 9 unique compounds (Table 4). Some of these compounds are known to exhibit antimicrobial, antifungal, and anticancer activities, and belong to different chemical groups, including amides, esters, ethers, alkaloids, amines, pyrimidines, ketones, hydrides, pyridazines, imidazoles, and silanes.

**Table 2:** List of unique bioactive compounds identified from the 7-day-old culture extract of *Streptomyces* sp. VITGV100 elicited with 0.5% dimethyl sulfoxide

Sr. No.	Compound name	Rt (min)	Area (%)	Molecular formula	Molecular mass	Application	Nature
1	Propanamide, N-(2,6-dimethylphenyl)-3-(4-morpholyl)-	10.242	0.56	C_15_H_22_N_2_O_2_	262.35	–	Amide
2	(1-Ethyl-2-methylpropyl) dimethylamine	13.162	0.29	C_8_H_19_N	129.24	Anti-inflammatory property	Amine
3	6-Amino-5-butylamino-4(3H)-pyrimidinone	14.663	0.42	C_8_H_14_N_4_O	182.22	–	Pyrimidine
4	Cyclohexanecarboxylic acid, 3-methylbut-2-yl ester	16.509	2.40	C_12_H_22_O_2_	198.3019	Antimicrobial property	Ester
5	Fumaric acid, 2,6-dimethoxyphenyl 3-methylbut-2-en-1-yl ester	17.431	2.22	C_17_H_20_O_6_	320.3371	–	Ester
6	L-leucine, N-cyclopropylcarbonyl-, methyl ester	18.119	1.12	C_11_H_19_NO_3_	213.27	Antimicrobial property	Ester
7	Isopropyl 3-methylorotate	20.988	1.23	C_9_H_12_N_2_O_4_	212.20	Fungicidal property	Ester
8	Glutaric acid, dodec-2-en-1-yl 2,6-dimethoxyphenyl ester	21.282	0.54	C_25_H_38_O_6_	434.5656	–	Ester
9	Benzoimidazol-1-yl-[4-(4-chloro-benzyloxy)-3-methoxy-phenyl]-methanone	22.054	4.57	C_15_H_12_N_2_O_2_	252.275	Antioxidant property	Ketone
10	Cyclopentathiazole	22.406	2.11	C_6_H_7_NS	125.191	Anti-inflammatory property	Heterocyclic
11	2-Fluorobenzoic acid heptadecyl ester	23.949	1.11	C_24_H_39_FO_2_	378.6	Antimicrobial property	Benzoic acid
12	2′-Hydroxy-5′-methylacetophenone, TMS derivative	28.060	1.59	C_9_H_10_O_2_	150.18	Antimicrobial and antifungal properties	Ketone

Rt – retention time, Sr. No. – serial number.

**Table 3:** List of unique bioactive compounds identified from the 14-day-old culture extract of *Streptomyces* sp. VITGV100 elicited with 0.5% dimethyl sulfoxide

Sr. No.	Compound name	Rt (min)	Area (%)	Molecular formula	Molecular mass	Application	Nature
1	Lacthydrazide	13.162	0.61	C_3_H_8_N_2_O_2_	104.11	Antimicrobial property	Hydride
2	3,3-Diacetyl-2,3,4,5-tetrahydro-2oxofuran	16.274	1.77	C_8_H_10_O_4_	170.16	Anticancer property	Ether
3	Cyclohexanecarboxylic acid, 3-methylbut-2-yl ester	16.500	4.07	C_12_H_22_O_2_	198.3019	–	Ester
4	3-Chloro-7H-imidazo[4,5-c] pyridazine	17.146	7.66	C_7_H_7_ClN_4_	182.61	Antimicrobial property	Pyridazine
5	L-Leucine, N-cyclopropylcarbonyl-, methyl ester	18.128	3.23	C_11_H_19_NO_3_	213.27	Antimicrobial property	Ester
6	L-Proline, N-valeryl-, decyl ester	18.312	11.47	C_20_H_37_NO_3_	339.5	Antimicrobial property	Ester
7	cis-4-Decene	18.899	0.25	C_10_H_20_	140.27	Antimicrobial property	–
8	DL-Norleucine, N-allyloxycarbonyl-, hexadecyl ester	19.495	0.28	C_26_H_49_NO_4_	439.67156	Larvicidal property	Ester
9	3-Azetidin-1-yl-propionic acid, methyl ester	20.091	0.70	C_7_H_13_NO_2_	143.19	Antibacterial and cytotoxic properties	Alkaloid
10	Thiazole, 4,5-dimethyl-2-propyl	21.391	0.59	C_8_H_13_NS	155.261	Antibacterial property	Heterocyclic
11	3-Aminooxypentanedioic acid, diethyl ester	23.958	1.26	C_9_H_17_NO_5_	219.23	–	Ester
12	1H-imidazole, 1-(1-naphthalenylmethyl)-	24.335	3.85	C_14_H_12_N_2_	208.26	Antibacterial and anti-inflammatory properties	Imidazole
13	N-m-tolyl-succinamic acid	24.788	1.26	C_11_H_13_NO_3_	207.23	Antibacterial property	Amide
14	Sebacic acid, 2,6-dimethoxy phenyl tridecyl ester	25.124	1.89	C_16_H_34_O_4_Si_2_	346.6098	–	Ester

Rt – retention time, Sr. No. – serial number.

**Table 4:** List of unique bioactive compounds identified from the 21-day-old culture extract of *Streptomyces* sp. VITGV100 elicited with 0.5% dimethyl sulfoxide

Sr. No.	Compound name	Rt	Area	Molecular formula	Molecular mass	Application	Nature
1	Thiophene, 2-butyl-5-propyl-	14.655	0.49	C_11_H_18_S	182.33	–	–
2	3-Dodecene, (E)-	15.989	0.44	C_12_H_24_	168.3190	Antifungal and antimicrobial properties	Alkene
3	3,3-Diacetyl-2,3,4, 5-tetrahydro-2-oxofuran	16.257	1.41	C_8_H_10_O_4_	170.16	Antibacterial property	Furanone
4	Cyclohexanecarboxylic acid, 3-methylbut-2-yl ester	16.492	2.55	C_12_H_22_O_2_	198.3019	Antifungal and antimicrobial properties	Ester
5	1,3-Benzodioxole-5-carboxylic acid	19.168	0.46	C_8_H_6_O_4_	166.1308	Antibacterial property	–
6	N-3-Diethylaminopropylsuccinimide	20.544	7.30	C_4_H_5_NO	115.09	Antimicrobial property	Amide
7	Fumaric acid, butyl 3-methylbut-3-enyl ester	20.980	0.91	C_13_H_20_O_4_	240.2955	Antimicrobial property	Ester
8	L-Proline, N-pivaloyl-, octyl ester	21.282	0.60	C_18_H_33_NO_3_	311.4595	–	Ester
9	Methyl tris(trimethylsiloxy)silane	28.547	1.62	C_10_H_24_O_6_Si	268.38	Antibacterial property	Silane

Rt – retention time, Sr. No. – serial number.

**Figure 2:**
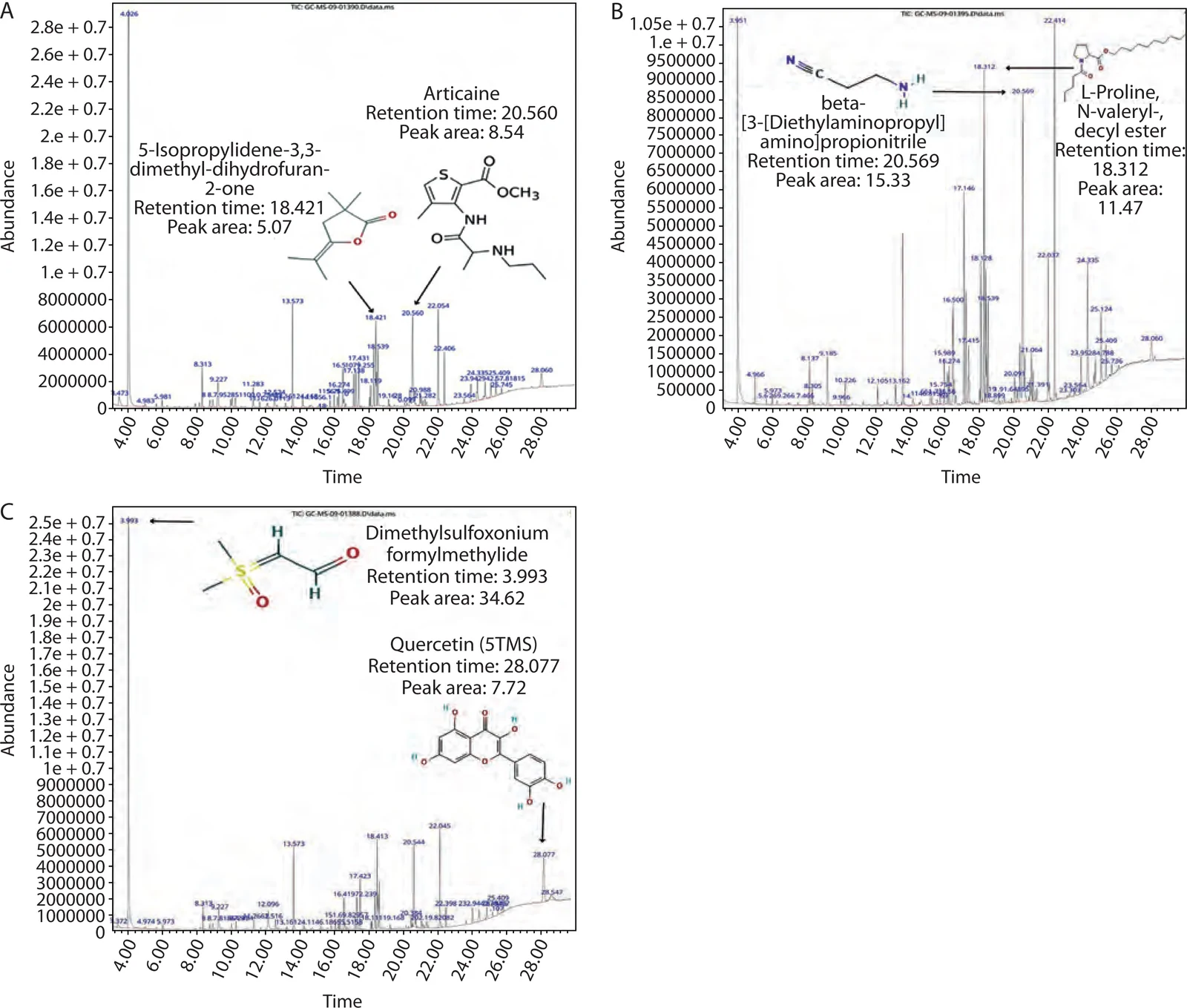
Gas chromatography–mass spectrometry chromatograms of ethyl acetate extracts from *Streptomyces* sp. VITGV100 treated with 0.5% dimethyl sulfoxide. (**A**) 7-day incubation showing major peaks for articaine (Retention [Rt]: 20.560 min, 8.54% area) and 5-isopropylidene-3,3-dimethyldihydrofuran-2-one (Rt: 18.421 min, 5.07% area). (**B**) 14-day incubation showing major peaks for beta-[3-[Diethylaminopropyl] amino] propionitrile (RT: 20.569 min, 15.3% area) and L-Proline, N-valeryl-, decyl ester (Rt: 18.312 min, 11.4% area). (**C**) 21-day incubation showing major peaks for dimethylsulfoxonium formylmethylide (Rt: 3.993 min, 34.62% area) and quercetin (5TMS) (Rt: 28.077 min, 7.72% area)

**Figure 3:**
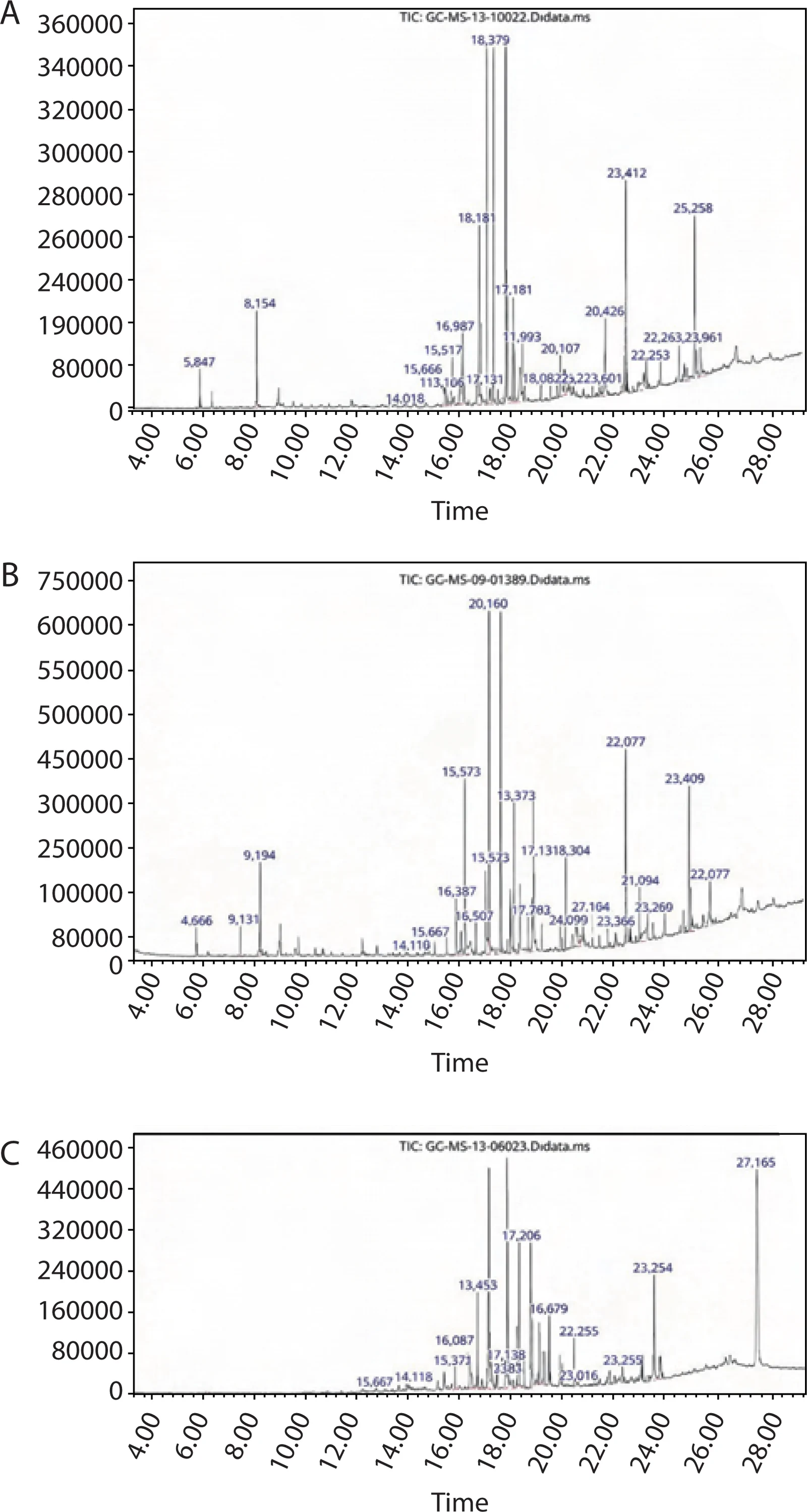
The gas chromatography-mass spectrometry (GC-MS) profiling of the control culture was performed on extracts collected on the 7^th^, 14^th^, and 21^st^ days of incubation

GC–MS analysis revealed distinct temporal variations in the metabolite profiles across different incubation timelines. In the 7-day-old culture, major peak areas were observed for articaine at 8.54% and 5-isopropylidene-3,3-dimethyl-dihydrofuran-2-one at 5.07% (Figure 2A). Similarly, in the 14-day-old culture, beta-[3-[diethylaminopropyl] amino] propionitrile and L-pro-line, N-valeryl-, decyl ester exhibited prominent peak areas at 15.3% and 11.47%, respectively (Figure 2B). Finally, in the 21-day-old culture, dimethylsulfoxonium formylmethylide and quercetin (5TMS) displayed peak areas at 34.62% and 7.72%, respectively (Figure 2C). These variations indicate a dynamic shift in the compound accumulation over incubation times.

The identified compounds encompassed a diverse range of chemical classes, including alkaloids, imidazoles, alkenes, furanones, silanes, amides, amines, pyrimidines, esters, ketones, heterocyclic compounds, and hydrides, many of which possess anti-inflammatory, antimicrobial, fungicidal, antioxidant, anticancer, larvicidal, and cytotoxic properties (Salama et al. 2022). These findings highlight the wide chemical diversity of the strain VITGV100, providing a comprehensive foundation for understanding its chemical composition and potential bioactivity. For further validation of antimicrobial activity, molecular docking simulations were performed.

Consistent with prior GC–MS investigations of actinobacterial secondary metabolites, this study aimed to identify and characterize the chemical compounds produced by the strain VITGV100. Through GC–MS analysis, we detected a range of unique chemical compounds with distinctive retention times. These previously unreported compounds were found exclusively in the VITGV100 strain when incubated with 0.5% DMSO for 7, 14, and 21 days. The compounds include propanamide, N-(2,6-dimethylphenyl)-3-(4-morpholyl)-, (1-ethyl-2-methylpropyl) dimethylamine, 6-amino-5-butylamino-4(3H)-pyrimidinone, fumaric acid, 2,6-dimethoxyphenyl 3-methylbut-2-en-1-yl ester, glutaric acid, dodec-2-en-1-yl 2,6-dimethoxyphenyl ester, benzoimidazol-1-yl-[4-(4-chlorobenzyloxy)-3-methoxy-phenyl]-methanone, lacthydrazide, and N-3-diethylaminopropylsuccinimide. Notably, several metabolites, which have previously been reported from plasma exosomes of ovarian cancer (Long et al. 2024) and ethanol extracts of *Euscaphis japonica* bark (Wei et al. 2020), were detected for the first time in *Streptomyces* sp. VITGV100. This suggests that the DMSO chemical elicitor effectively activated dormant biosynthetic pathways in this strain, leading to the synthesis of these unique metabolites.

The detection of lacthydrazide is particularly remarkable, given its classification as a siderophore-type compound with strong antimicrobial potential. Its expression, likely induced by DMSO-mediated activation of a BGC sharing 9% similarity with known siderophore clusters (Veilumuthu et al. 2022), highlights the capacity of chemical elicitation to uncover novel bioactive metabolites from *Streptomyces* sp. VITGV100.

During the 7-day incubation period, our GC–MS analysis revealed two major peaks with the highest area percentages of 8.54% and 5.07%. These peaks corresponded to articaine and 5-isopropylidene-3,3-dimethyldihydrofuran-2-one, with retention times of 20.560 and 17.138 min, respectively (Figure 2A). By day 14, we observed two major peaks at 15.33% and 11.47%, identified as -[diethylaminopropyl] amino] propionitrile and L-proline, N-valeryl-, decyl ester, with retention times of 20.569 and 18.312 min, respectively (Figure 2 B). At day 21, the profile was dominated by two major peaks at 34.62% and 7.72%, attributed to dimethylsulfoxonium formylmethylide and quercetin (5TMS), with retention times of 3.993 and 28.077 min, respectively (Figure 2C), which align with the retention times of 6 and 29.9 min, respectively, reported in previous studies (El-Moslamy et al. 2023; Yıldırım 2003). These results provide valuable insights into the temporal dynamics of secondary metabolite production by strain VITGV100. The observation of different peaks for *Streptomyces* culture extracts from different incubation times could stem from compound degradation or transformation, growth phase transitions, metabolic activities, pathway inductions, or nutrient availability changes (El-Moslamy et al. 2023).

### Molecular docking analysis

Molecular docking using AutoDock tools revealed the promising interaction of lacthydrazide compound with the target proteins implicated in antimicrobial activity (Table 5). Two- and three-dimensional images of the complexes were visualized using LigPlot and PyMOL, respectively. The docking analysis demonstrated favorable binding energies and interactions, including hydrogen bonding with key residues within the protein binding sites. Remarkably, most of the interacting residues overlapped with the binding sites of co-crystallized ligands on target proteins (indicated in bold in Table 6). Lacthydrazide exhibited high binding affinity toward all the target proteins – *E. coli* Z-ring-associated protein (PDB ID: 5IMJ), *P. aeruginosa* OXA10 (PDB ID: 4WZ5), *S. aureus* TyrRS (PDB ID: 1JIJ), and *B. subtilis* DLTA NRPS adenylation domains (PDB ID: 3E7X) – highlighting its potential as a broad-spectrum inhibitor. The specific binding interactions of lacthydrazide with these proteins are illustrated in Figure 4, while interactions with additional potent complexes are presented in Supplementary Figures 1–4.

**Table 5:** Molecular docking results of siderophore-type lacthydrazide from ethyl acetate extract

Compound name	Rt (min)	Area (%)	Molecular formula	Molecular mass	Application	Nature	Type	Molecular structure
Lacthydrazide	13.162	0.61	C_3_H_8_N_2_O_2_	104.11	Antimicrobial property	Hydride	Siderophore	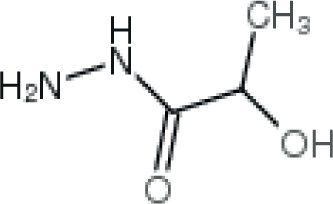

Rt – retention time.

**Table 6:** Binding energies and interacting residues (bold denotes important residues in target proteins interacting with potent compound)

Target proteins (PDB ID)	Binding energy [kcal/mol]	Interacting residues
	Lacthydrazide	Lacthydrazide
*Escherichia coli* Z-ring-associated protein (5IMJ)	–4.9	Arg 45, His 42, Lue 41, Glu 49, Asn 46, Cys 131
*Pseudomonas aeruginosa* OXA10 (4WZ5)	–4.8	Ser 115, Trp 154, Ser 67, Val 117, Phe 120, Phe 69
*Staphylococcus aureus* TyrRS (1JIJ)	–4.9	Asn 124, Thr 175, Gln 174, Tyr 36, Gln 190, Tyr 170, Asp 177, Lue 70, Asp 40
*Bacillus subtilis* DLTA: implicated for the reaction mechanism of NRPS adenylation domains (3E7X)	–5.1	Gly 269, Thr 292, Leu 272, Asn 291, Glu 270, Cys 268, Tyr 293, Val 320, Val 271

Bold indicates residues that overlap with the binding sites of co-crystallized ligands on the respective target proteins. NRPS – non-ribosomal peptide synthetase, PDB – Protein Data Bank.

**Figure 4:**
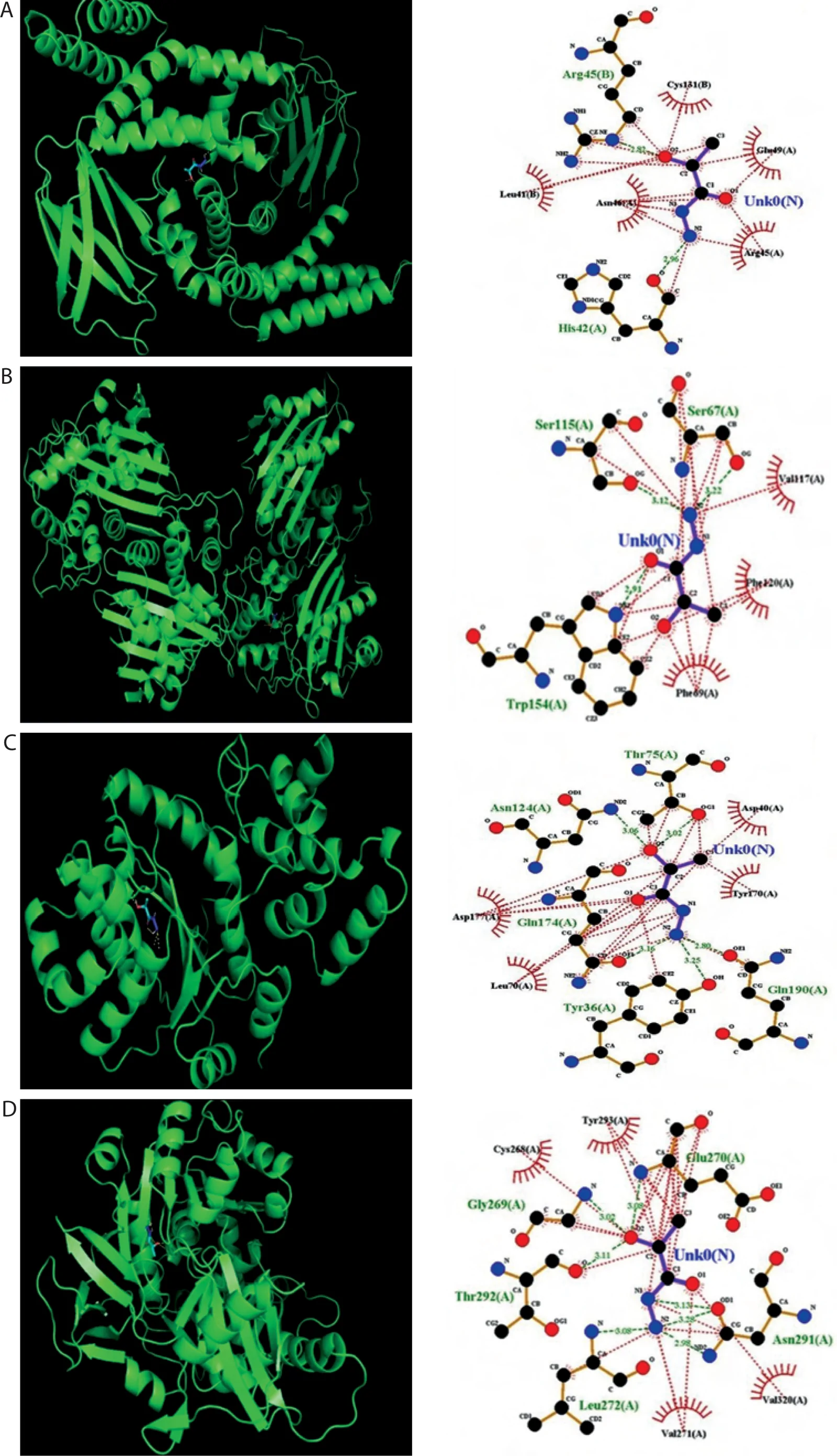
(**A**) Illustrates the binding interactions between the lacthydrazide complex with the target protein *E. coli* Z-ring-associated protein. Similarly, (**B**) demonstrates the binding interactions of lacthydrazide complex with target protein *Pseudomonas aeruginosa* OXA10. Additionally, (**C**) represents the binding interactions of the lacthydrazide complex with the target protein *Staphylococcus aureus* TyrRS, while (**D**) depicts the binding interactions of the lacthydrazide complex with the target protein *Bacillus subtilis* DLTA

The crystal structures and the corresponding Lig-Plot results are shown in Figure 4. Figure 4A illustrates the binding interactions between the lacthydrazide complex and the *E. coli* Z-ring-associated protein. Similarly, Figure 4B demonstrates the binding interactions of lacthydrazide with *P. aeruginosa* OXA10. Figure 4C presents the binding interactions of lacthydrazide with *S. aureus* TyrRS, and Figure 4D depicts the interactions with *B. subtilis* DLTA.

Molecular docking optimizes the conformation and orientation of receptors and ligands to minimize binding free energy (Mohanty and Mohanty 2023). Although in vitro experiments remain essential to validate ligand activity, molecular docking presents a reliable method for predicting receptor–ligand interactions (Fan et al. 2019). Here, the results were evaluated based on docking poses and specific protein–ligand interactions. Typically, lower binding energy (–4.8 to –5.1 kcal/mol) correlated with better stability of the complex (Dolgonosov 2017), suggesting favorable interactions with the target proteins. Crucially, molecular docking provides purely a theoretical estimation of binding potential and does not account for dynamic factors such as solvent effects, conformational flexibility, and entropic contributions. Therefore, these results should be interpreted cautiously and validated through further in vitro and in vivo studies. Nevertheless, the present study identified lacthydrazide as a potent inhibitor for target proteins, corroborated by significant binding energy and favorable interactions.

Although lacthydrazide has been previously identified in *Streptomyces* (Long et al. 2024), a molecular docking investigation on this compound had not been performed. The compound exhibited relatively low binding energies with all evaluated target proteins. Its interaction predominantly involves residues located within binding sites, analogous to those interacting with co-crystallized receptor ligands documented in the RCSB database (Charbe et al. 2024). The affinity between the ligand and the receptor protein is influenced by factors such as the number and spatial distances of hydrogen bonds, as well as their binding energy. In particular, certain poses exhibit hydrogen bonding and hydrophobic interaction with amino acid residues within the active site of essential bacterial membrane proteins. Therefore, molecular docking techniques provide valuable insights to validate the potency of these metabolites. Despite inherent limitations, such as a dependence on static structures and oversimplified scoring algorithms, docking offers important preliminary data regarding ligand–protein interactions. As a result, the study’s modest binding affinities should be regarded with caution and confirmed through further experimental research.

## Conclusions

The discovery of novel bioactive compounds is imperative to overcome the challenge of multidrug-resistant infections. *Streptomyces* sp. VITGV100 (MCC 4961) is emerging as a promising source of diverse antimicrobial agents, including the secondary metabolite lacthydrazide. Through the strategic application of chemical elicitors alongside varying incubation times, we effectively enhanced metabolite production in *Streptomyces* sp. VITGV100. Notably, this strain showed remarkable antimicrobial efficacy against *E. coli, P. aeruginosa, S. aureus*, and *B. subtilis*. This elicitation strategy not only sheds light on the bioactivity of the metabolites produced but also highlights the pathways amenable to large-scale production. In addition, subsequent molecular docking analysis confirmed the robust inhibitory activity of lacthydrazide against essential bacterial target proteins, highlighting the promising antimicrobial properties of *Streptomyces* sp. VITGV100 and its potential role in next-generation antibiotic production.

## Supplementary Material


