# Supplementary material for: GC-MS identification of a lacthydrazide-class siderophore produced by Streptomyces sp. VITGV100: its antimicrobial activity and molecular docking
Source: BioTechnologia (Pozn). 2026 Sep 21;107(3):247–60. doi: 10.5114/bta/224355 (PMC13725489; doi:10.5114/bta/224355)
Supplement: Supplementary file 1 [file BTA-107-3-224355-s1.pdf]

Supplementary data for antimicrobial analysis statistics

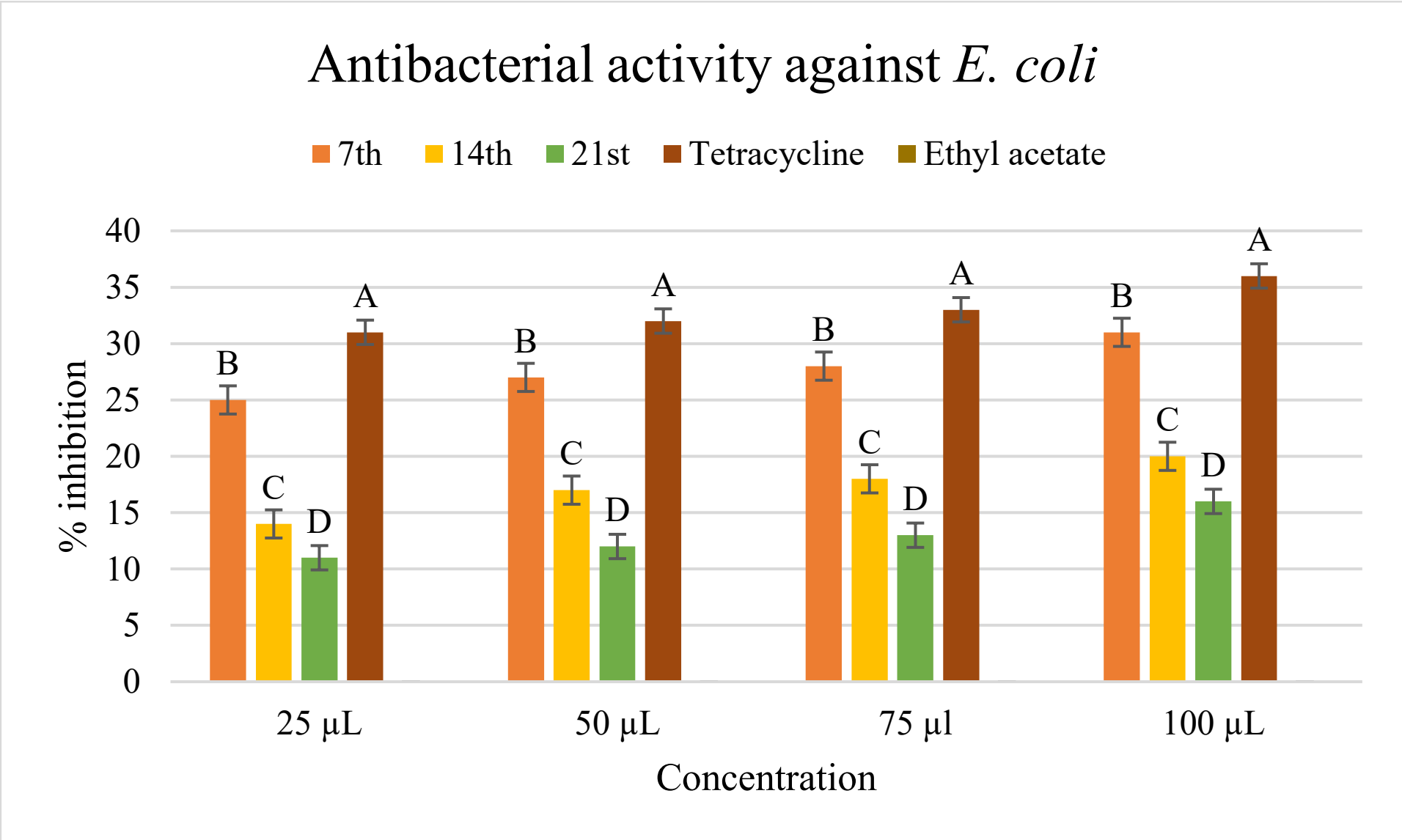

Figure S1. Mean zone of inhibition by the bar graph represents the highest zone of inhibition 31 mm, against *Escherichia coli* at 100 µl/ml at the different incubation and the distinct letters represent statistically significant differences between all incubation periods and the control ($p < 0.05$)

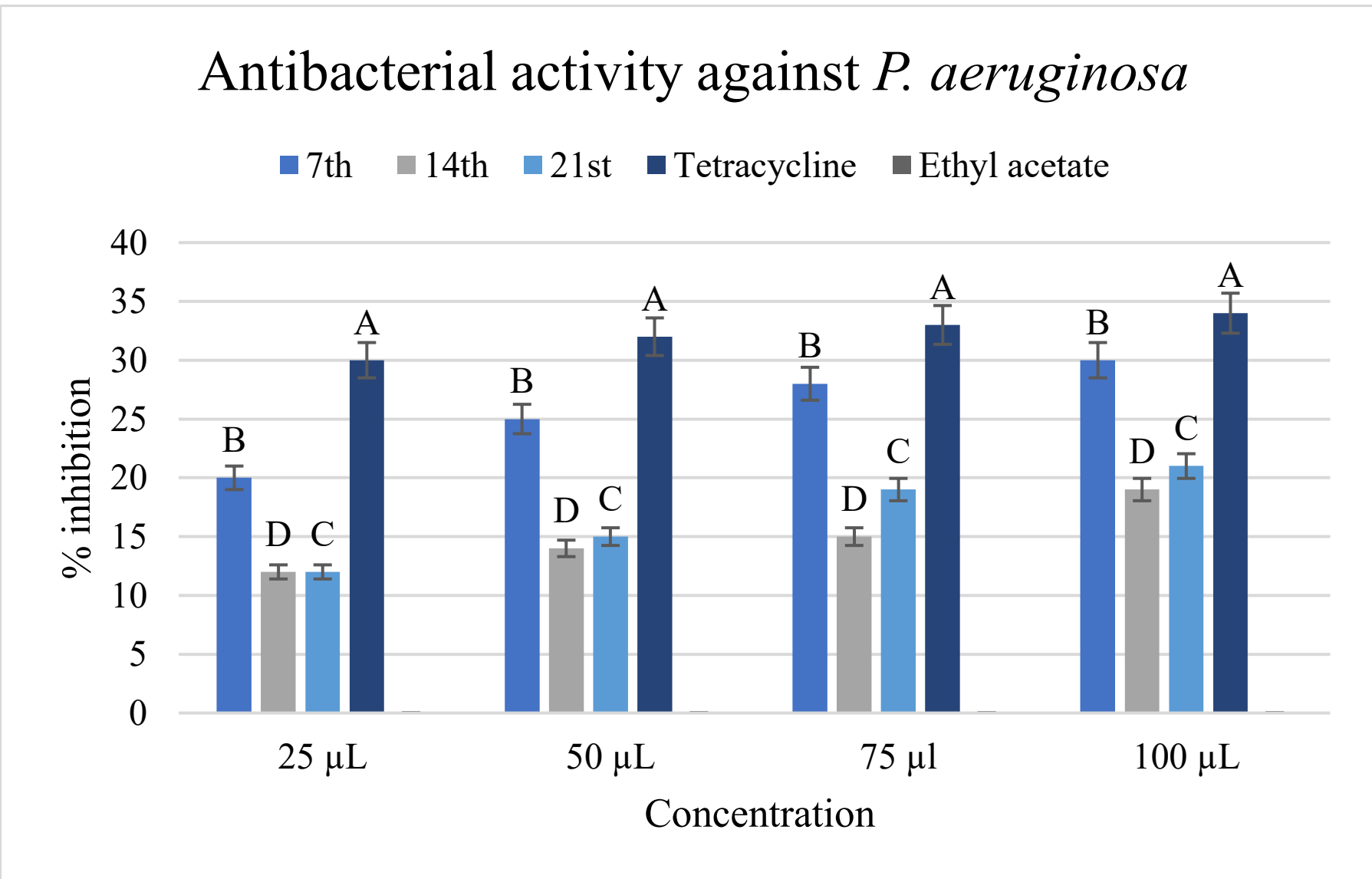

Figure S2. Mean zone of inhibition by the bar graph represents the highest zone of inhibition, 30 mm against *Pseudomonas aeruginosa* at 100 µl/ml at the different incubation and the distinct letters represent statistically significant differences between all incubation periods and the control ($p < 0.05$)

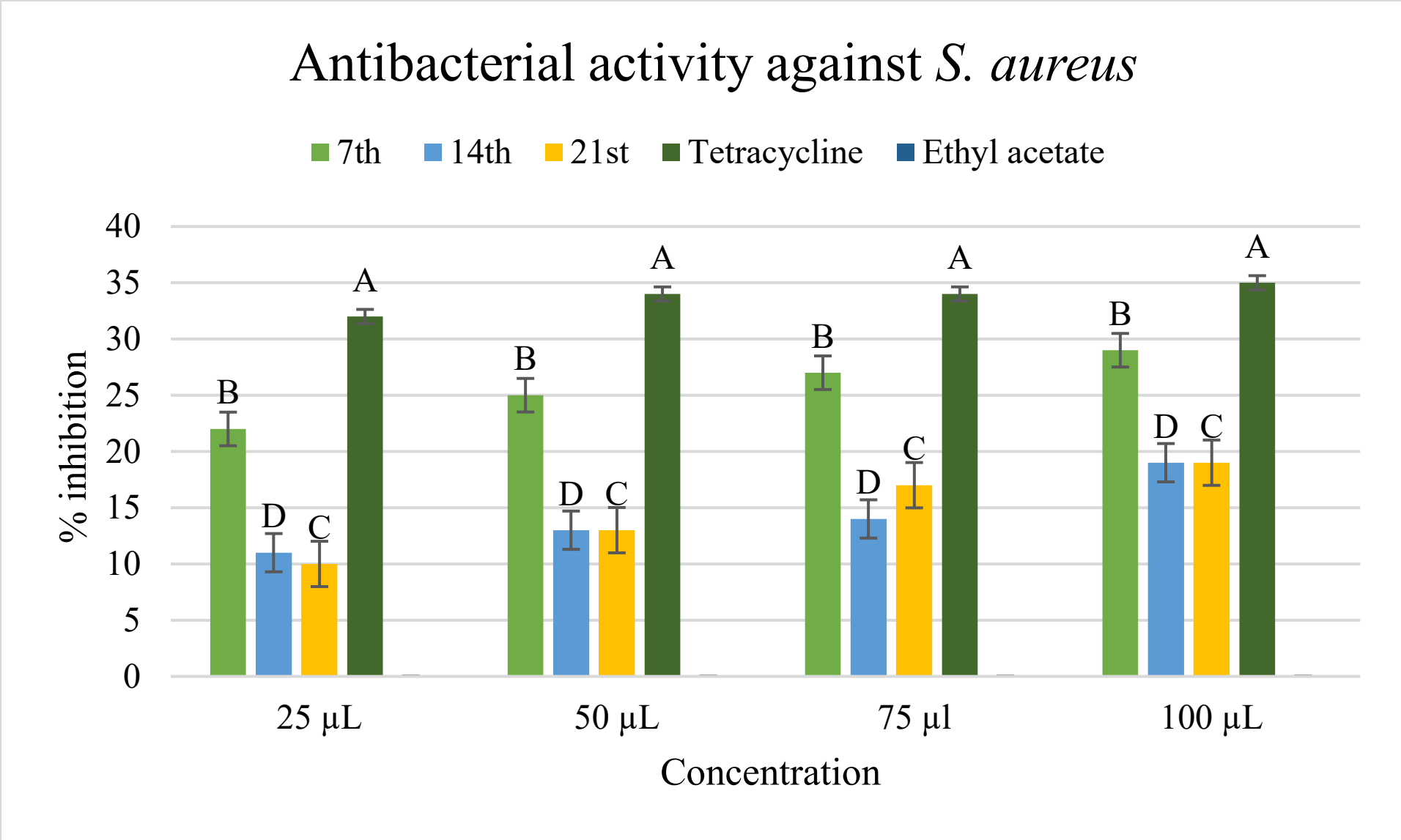

Figure S3. Mean zone of inhibition by the bar graph represents the highest zone of inhibition, 29 mm against *Staphylococcus aureus* at 100 µl/ml at the different incubation and the distinct letters represent statistically significant differences between all incubation periods and the control ($p < 0.05$)

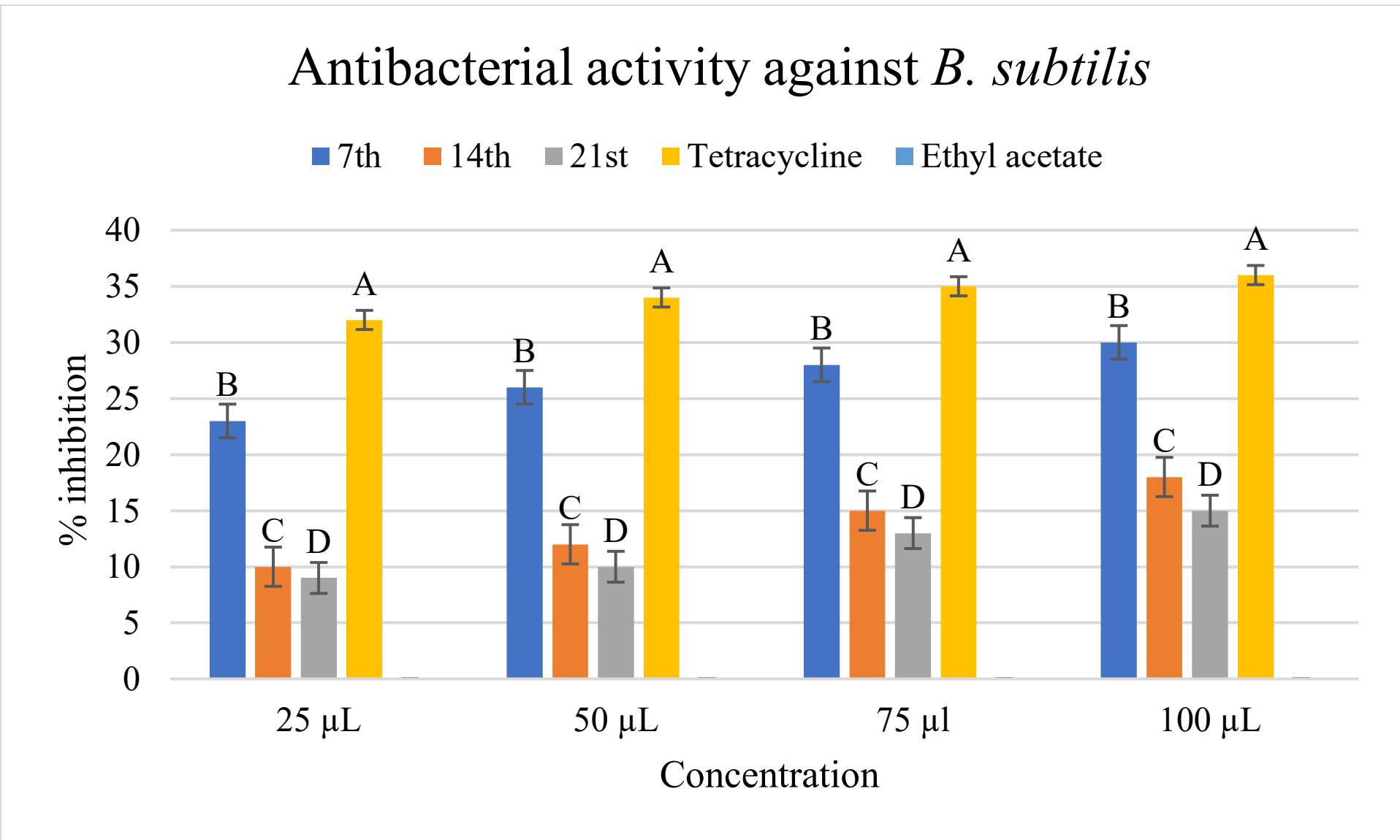

Figure S4. Mean zone of inhibition by the bar graph represents the highest zone of inhibition 30 mm, against *Bacillus subtilis* at 100 µl/ml at different incubation and the distinct letters represent statistically significant differences between all incubation periods and the control ($p < 0.05$)
